# Development of a predictive model based on aqueous cytokines data for response to anti-VEGF therapy in diabetic macular edema

**DOI:** 10.1186/s40662-026-00479-z

**Published:** 2026-03-13

**Authors:** Fuhua Yang, Yi Gong, Xiaoying Pan, Jinzhi Zhao, Rongguo Yu, Liangzhang Tan, Emmanuel Eric Pazo, Xiaomin Zhang, Xiaorong Li

**Affiliations:** Tianjin Key Laboratory of Retinal Functions and Diseases, Tianjin Branch of National Clinical Research Centre for Ocular Disease, Eye Institute and School of Optometry, Tianjin Medical University Eye Hospital, No. 251, Fukang Road, Nankai District, Tianjin, 300384 China

**Keywords:** Diabetic macular edema, Anti-VEGF, Cytokines, Predictive model

## Abstract

**Background:**

Intravitreal anti-vascular endothelial growth factor (VEGF) agents improve visual acuity in diabetic macular edema (DME). However, resistance, non-response, or recurrence occurs in many patients. Predictive biomarkers for anti-VEGF response are lacking. We aim to identify cytokine markers predictive of anti-VEGF response and elucidate cytokines involved in poor-response DME pathogenesis.

**Methods:**

A luminex assay was carried out to measure the concentration of cytokines in the aqueous humor. A predictive model based on baseline cytokines was constructed in a discovery set that comprised 46 responders and 20 non-responders. In addition, an analysis of baseline cytokines of 15 responders and 12 non-responders was conducted as a validation set. The performance of the nomogram was determined using the area under the receiver operating characteristic curve (AUC) and Hosmer–Lemeshow goodness of fit test.

**Results:**

Baseline concentrations of angiogenesis-related cytokines VEGF (*P* < 0.0001), placenta growth factor (PIGF) (*P* < 0.0001), angiopoietin-2 (Ang-2) (*P* < 0.0001), inflammatory factor interleukin-6 (IL-6) (*P* < 0.0001), IL-8 (*P* < 0.0001), chemokine monocyte chemoattractant protein-1 (MCP-1) (*P* < 0.0001), and adhesion factor intercellular adhesion molecule-1 (ICAM-1) (*P* < 0.001) were significantly increased compared to controls. The prediction nomogram model included five baseline cytokines: VEGF, IL-6, Ang-2, MCP-1, and ICAM-1 were constructed. The AUC for the discovery set was 0.85 (95% CI: 0.74–0.96) and for the internal validation was 0.84, indicating that the prediction model has good predictive accuracy. The Hosmer–Lemeshow goodness of fit test showed good model calibration (*P* = 0.295). The levels of Ang-2 (*P* = 0.0042), IL-6 (*P* < 0.0001), IL-8 (*P* < 0.0001), MCP-1 (*P* < 0.0001), and PIGF (*P* < 0.0001) were still significantly increased at the 6-month timepoint after multiple injections of anti-VEGF drugs for non-response group patients.

**Conclusion:**

The baseline cytokine-based model helped to assess the individual probability of response to anti-VEGF therapy. There are cytokines beyond VEGF that are involved in the pathogenesis of DME, therapeutic regimens targeting these cytokines may improve the visual acuity and reduce macular edema.

**Supplementary Information:**

The online version contains supplementary material available at 10.1186/s40662-026-00479-z.

## Background

Diabetic retinopathy (DR) is the most common type of diabetic eye disease, which is the leading cause of blindness in the working-age population with type 2 diabetes [1, 2]. Diabetic macular edema (DME) is a severe eye condition caused by DR. Macula is a part of the retina and is needed for sharp, central vision. The blood vessels in the retina leak fluid into the macula, which could cause DME, the most common cause of visual loss in diabetic patients [3].

Currently, the treatment options for DME include laser treatment, steroid treatment, anti-VEGF injections, and vitrectomy surgery. Anti-vascular endothelial growth factor (VEGF) therapy has become the first line treatment [4, 5] as it can prevent the leakage from the abnormal blood vessels in the eye by blocking the development of new blood vessels, and thus decrease edema and improve the visual acuity of DME patients. Although anti-VEGF agents have revolutionized the treatment of DME, a significant proportion of patients continue to exhibit suboptimal or limited responses despite multiple intravitreal injections [5, 6]. Variability in therapeutic response to anti-VEGF agents remains a significant clinical challenge in the management of DME. Early identification of patients who exhibit poor or inadequate response is critical, as it enables timely transition to alternative therapeutic regimens that may yield better outcomes. Moreover, accumulating evidence suggests that persistent or suboptimal response to anti-VEGF treatment, despite multiple intravitreal injections, indicates the involvement of additional molecular pathways beyond VEGF in the pathogenesis of DME [3, 6].

The objective of this study is to assess biomarkers that can be used to predict how patients will respond to anti-VEGF therapy. Additionally, we hope to validate the involvement of cytokines, rather than VEGF, in the development of DME. To investigate the changes in specific cytokine levels in the aqueous humor of DME patients following intravitreal anti-VEGF injections, we compared samples collected before and after treatment. Therapeutic regimens targeting these cytokines, rather than VEGF, may improve visual acuity and reduce macular edema in patients with DME.

The baseline cytokine-based nomogram model was constructed to assess the individual probability of response to anti-VEGF therapy and may assist physicians in making better treatment decisions.

## Methods

### Participants and study design

This study was approved by the Institutional Ethics Committee of Tianjin Medical University Eye Hospital (No. 2017KY-01) and complied with the Declaration of Helsinki. All participants provided written informed consent. This consent contained detailed descriptions of the repeated paracentesis procedures, covering their purpose and potential risks, such as hyphema, infection, intraocular pressure (IOP) elevation, and iatrogenic injury. Potential complications following each paracentesis procedure were systematically monitored and recorded throughout the study. This was a single-center study conducted at Tianjin Medical University Eye Hospital, and all participants were enrolled at this institution. All enrolled DME patients were Han Chinese and had type 2 diabetes in this study, and the DME patients were all with central macular thickness (CMT) > 250 μm in the study eyes on the optical coherence tomography (OCT). Eyes with severe vitreous hemorrhage, tractional retinal detachment, glaucoma, cataract, uveitis or received intraocular surgery within the past 6 months were excluded. The control group comprised patients undergoing cataract surgery with no diabetes and no other ocular diseases. All patients received a comprehensive ophthalmologic evaluation including best-corrected visual acuity (BCVA) recorded as logarithm of the minimum angle of resolution (logMAR) and IOP. Demographic and clinical information, including sex, age, duration of diabetes mellitus, BCVA, and CMT, were collected at baseline.

All eligible eyes were given the initial anti-VEGF drug ranibizumab injection at a dose of 0.5 mg in 0.05 mL (Lucentis) and continued to receive monthly injections for three consecutive months and retreatment based on disease activity [pro re nata (PRN)] was administered from Month 3 to Month 6 for the remainder of the study. The criteria for PRN include a decrease in BCVA of >5 letters or an increase in CMT of >50 μm as shown by OCT. Our study was a single-phase, retrospective observational cohort designed to correlate cytokine levels with treatment response. It did not involve separate discovery and validation phases. Instead, bootstrap internal validation was performed on the entire cohort. Participants were managed as follows: baseline assessment and sample collection prior to the first anti-VEGF injection. The clinical course was monitored by measuring CMT and BCVA at 4-week intervals throughout the 24-week study period. Eyes were categorized into distinct responder groups using the following criteria: (ⅰ) rapid responders were those eyes in which improvement in the BCVA > 5 letters one month after the first intravitreal injection; (ⅱ) slow responders were those eyes who met these criteria between Months 2 and 6; (ⅲ) refractory responders were those eyes that improvement in the BCVA < 5 letters by 6 months of 3 + PRN treatment. The eyes were classified into response groups (i and ii), and non-response group (iii).

### Sample collection and measurement

**Fig. 1:**
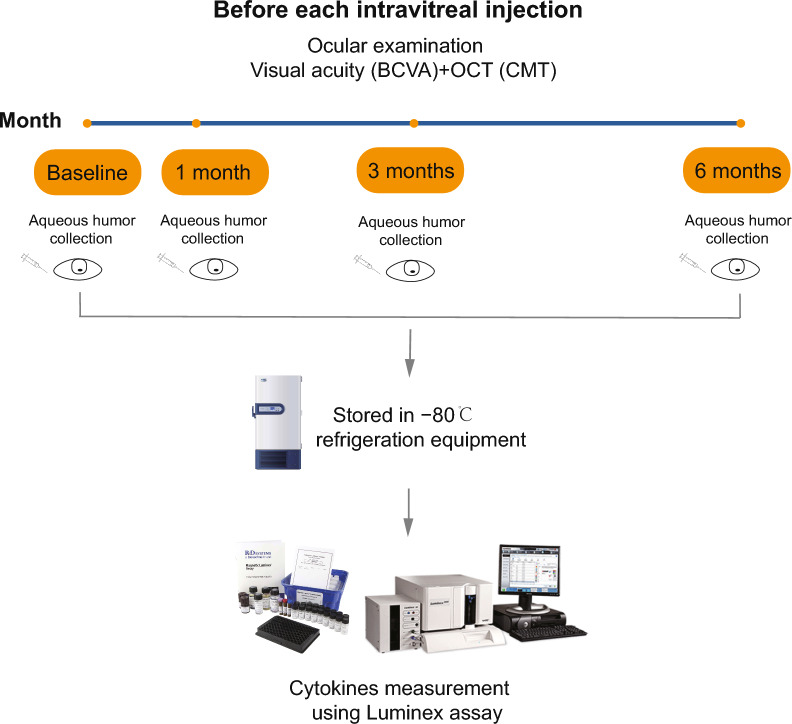
Study design. Aqueous fluid specimens for cytokine analysis were obtained before an initial injection and at 1, 3, 6 months post-injection, and stored at −80 ℃ until further analysis. BCVA, best-corrected visual acuity; OCT, optical coherence tomography; CMT, central macular thickness

Aqueous fluid specimens for cytokine analysis were obtained before an initial injection and at 1, 3, and 6 months after the injection (Fig. 1). Aqueous fluid from age-matched cataract patients were used as control samples. Only one eye per patient was included in the study. Aqueous humor is typically collected between 8:00 a.m. and 10:00 a.m. The aqueous fluid samples were immediately placed in sterile tubes and stored at −80 ℃ for 6 to 12 months until further analysis. Prior to the Luminex analysis, samples were thawed only once to prevent protein degradation from multiple freeze–thaw cycles.

Multiplex immunoassay of all aqueous samples was carried out for candidate cytokines. The premixed multi-analyte kit (LXSAHM-01 and LXSAHM-07, R&D Systems, USA) included VEGF, placenta growth factor (PIGF), angiopoietin-2 (Ang-2), interleukin-6 (IL-6), IL-8, tumor necrosis factor-alpha (TNF-α), monocyte chemoattractant protein-1 (MCP-1), and intercellular cell adhesion molecule-1 (ICAM-1) (Supplementary Table S1) was used according to the manufacturer’s instructions. Each sample was assayed in two replicate wells. Briefly, 50 μL of aqueous sample incubated with color-coded magnetic beads coated with capture antibodies. Biotinylated detection antibodies were then added, followed by an incubation with streptavidin–phycoerythrin. All measurements were performed on the Luminex 200 instrument.

### Statistical analysis

Log transformation helps make the cytokine concentrations data approximate a normal distribution, and log transformation allows more sensitive parametric testing. To enhance the efficacy of statistical tests and improve the accuracy, robustness, and interpretability of the regression model, the data were log-transformed. Cytokine concentrations below the limit of detection (LOD) were replaced with LOD/√2 to allow for log-transformation and subsequent analysis. After log transformation, the Shapiro–Wilk’s test was used to assess normality. To investigate the changes in aqueous humor cytokine levels in DME patients, we compared aqueous cytokine levels at baseline vs. control samples. Data was analyzed using the *t*-test for normally distributed variables or the Mann–Whitney U test for those not meeting normality assumptions (GraphPad Prism 9.0). For multiple comparisons, we performed corrections using the Benjamini–Hochberg false discovery rate (FDR) method. For the primary cytokine of interest (VEGF), we conducted a post-hoc power analysis using G*Power software based on the observed effect size, which indicated that the study had approximately 92.7% power to detect the observed difference at a significance level of 0.05. The association between baseline aqueous cytokine levels and different responses to anti-VEGF drugs treatment was estimated using Spearman correlation analysis (GraphPad Prism 9.0).

Features associated with therapy response were selected using the least absolute shrinkage and selection operator (LASSO) logistic regression, implemented via the “glmnet” package (R software version 4.3.3). Given the limited sample size, a threefold cross-validation scheme was employed. To maximize the use of information in the data and avoid omitting potential predictive features, we selected the lambda value that minimizes the cross-validation error (λmin). These selected features with the clinical covariates including age, baseline BCVA, baseline CMT, glycated hemoglobin A1c (HbA1c) and diabetes duration, were incorporated into a standard logistic regression model to develop a prediction score system. Subsequently, a baseline cytokine-based nomogram was constructed using the “rms” package to display the model. Given the relatively small sample size in this study, we performed internal validation using the bootstrap method with 500 resamples. The receiver operating characteristic (ROC) curves, area under the ROC curve (AUC), sensitivity and specificity (pROC package) were used to evaluate the discriminative performance of the model. To determine the optimal threshold for the model, the Youden Index was calculated, maximizing the sum of sensitivity and specificity. The model’s calibration was assessed using a calibration curve (rms package) and the Hosmer–Lemeshow goodness-of-fit test. and the potential of the model to inform clinical decisions was then quantified using decision curve analysis (DCA) (rmda package). To facilitate the prediction model in the clinical practice, an interactive online dynamic nomogram application was constructed using Shiny. A *P* value < 0.05 indicates statistical significance.

To characterize the dynamics of functional improvement, we performed a survival analysis using time to first functional response (defined as a gain of > 5 letters) as the endpoint. This approach allows for visualizing the cumulative response rate over time and identifying patterns of rapid versus delayed response. The Kaplan–Meier method (survival package) was employed to calculate the median response time and landmark cumulative response rates, and to plot the corresponding Kaplan–Meier curves.

## Results

### Elevated concentrations of vascular growth factors and inflammation-related cytokines were detected in DME patients

**Fig. 2:**
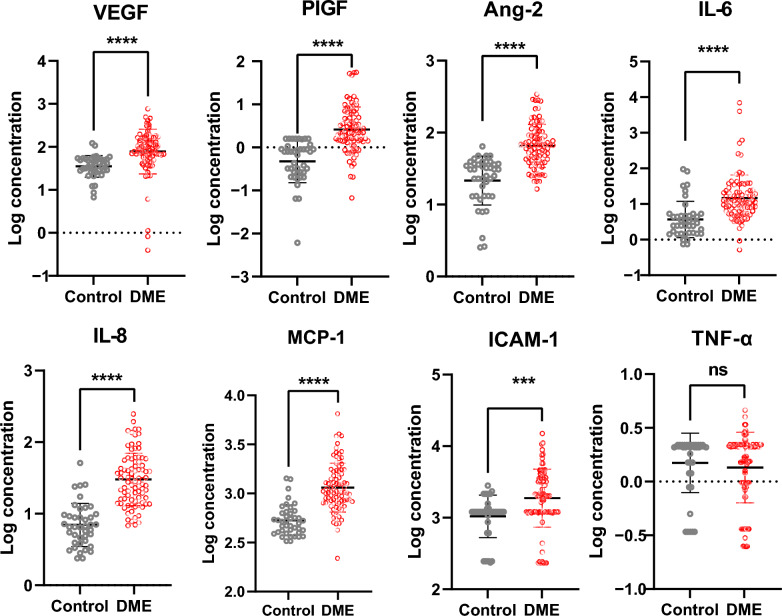
Baseline aqueous cytokine level was measured for diabetic macular edema (DME) patients. Baseline aqueous humor was collected from patients with DME (n = 85) and cataract patients (n = 41). The selected cytokine levels were measured using Luminex assay. Cytokine data were log-transformed, Mann–Whitney U test was used. ***P < 0.001, ****P < 0.0001, ns, non-significant; Ang-2, angiopoietin-2; ICAM-1, intercellular adhesion molecule-1; IL-6, interleukin-6; IL-8, interleukin-8; MCP-1, monocyte chemoattractant protein-1; PIGF, placenta growth factor; TNF-α, tumor necrosis factor-alpha; VEGF, vascular endothelial growth factor

The study recruited 85 DME patients (Table 1) and 41 age-matched controls (age 56.4 ± 11.6 years vs. 59.7 ± 8.4 years, *P* = 0.14), and baseline aqueous was obtained to measure the concentration of selected cytokines. Compared to controls, elevated cytokines including angiogenesis-related cytokines VEGF (logVEGF 1.9 ± 0.5 vs. 1.5 ± 0.3, *P* < 0.0001), PIGF (logPIGF 0.4 ± 0.5 vs. −0.3 ± 0.5, *P* < 0.0001), and Ang-2 (logAng-2 1.8 ± 0.3 vs. 1.3 ± 0.3, *P* < 0.0001), inflammatory factor IL-6 (logIL-6 1.2 ± 0.6 vs. 0.6 ± 0.5, *P* < 0.0001) and IL-8 (logIL-8 1.5 ± 0.4 vs. 0.8 ± 0.3, *P* < 0.0001), chemokine MCP-1 (logMCP-1 3.1 ± 0.2 vs. 2.7 ± 0.2, *P* < 0.0001), and adhesion factor ICAM-1 (logICAM-1 3.3 ± 0.4 vs. 3.0 ± 0.3, *P* < 0.001) were identified in DME patients (Fig. 2 and Supplementary Table S2).

**Table 1:** Basic clinical characteristics of diabetic macular edema (DME) patients

Characteristics	Values
Demographics (n = 85)	
Age (years)	56.4 ± 11.6
Sex, n (%)	
Male	51 (60)
Female	34 (40)
Best-corrected visual acuity (logMAR)	0.71 ± 0.49
CMT (μm)	449.72 ± 117.11
HbA1c (%)	7.65 ± 1.35
Duration of diabetes (years), n (%)	
< 5	21 (24.7%)
5 to 10	21 (24.7%)
> 10	43 (50.6%)

*CMT* = central macular thickness; *HbA1c* = glycated hemoglobin A1c

### Higher baseline VEGF, PIGF, Ang-2, IL-8, and ICAM-1 levels were detected in response group versus the non-response group

**Fig. 3:**
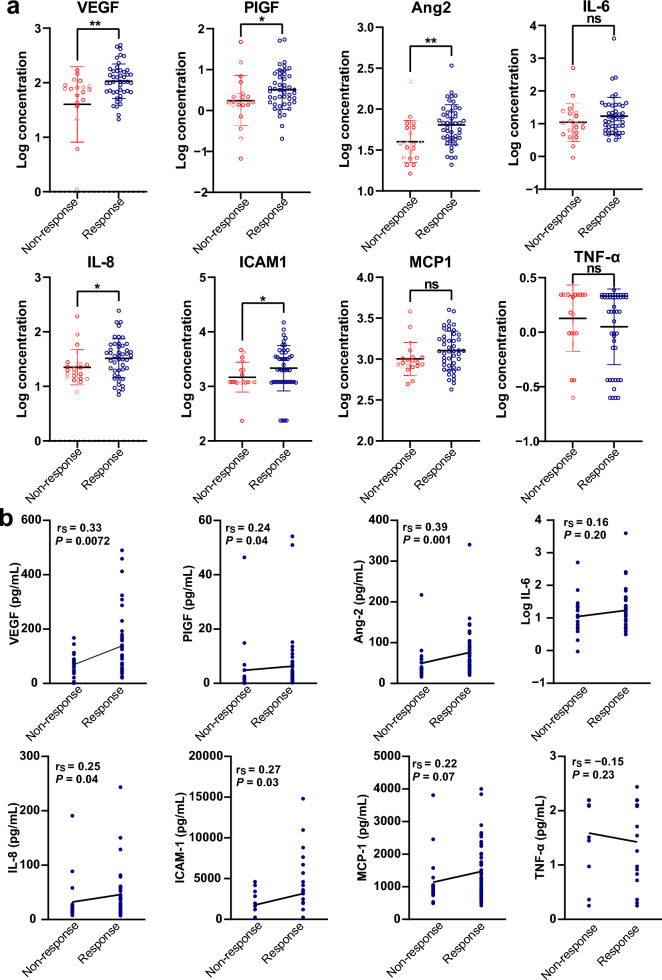
Cytokine levels in baseline aqueous. **a** Baseline aqueous was obtained from response (n = 46) and non-response groups (n = 20) and measured using the Luminex assay. Cytokine data were log-transformed. *P < 0.05, **P < 0.01, ns, non-significant. **b** Correlation between cytokines’ baseline levels and response to anti-VEGF therapy. Spearman correlation analysis was conducted to identify the correlation between cytokines and response to anti-VEGF therapy. The r_s_ coefficient of correlation and *P* values are shown. Ang-2, angiopoietin-2; ICAM-1, intercellular adhesion molecule-1; IL-6, interleukin-6; IL-8, interleukin-8; MCP-1, monocyte chemoattractant protein-1; PIGF, placenta growth factor; TNF-α, tumor necrosis factor-alpha; VEGF, vascular endothelial growth factor

We identified cytokines other than VEGF that were elevated in DME patients. A serial analysis of the cytokines across response and non-response groups was subsequently conducted. Of the 66 studied eyes with follow-up BCVA and CMT data, 69.7% (46/66) were responsive, and 30.3% (20/66) were non-responsive (Table 2). No procedure-related complications were observed during or after any of the repeated paracenteses performed in the non-response group. At baseline, responsive eyes have higher VEGF (logVEGF 2.0 ± 0.3 vs. 1.6 ± 0.7, *P* = 0.0075), PIGF (logPIGF 0.5 ± 0.5 vs. 0.2 ± 0.6, *P* = 0.0498), Ang-2 (logAng-2 1.8 ± 0.2 vs. 1.6 ± 0.3, *P* = 0.0012), IL-8 (logIL-8 1.5 ± 0.4 vs. 1.3 ± 0.3, *P* = 0.0446) and ICAM-1 (logICAM-1 3.3 ± 0.4 vs. 3.2 ± 0.3, *P* = 0.0286) concentration compared to non-responsive eyes (Fig. 3a and Supplementary Table S3).

**Table 2:** Baseline characteristics for responders and non-responders

Characteristic	Responders (n = 46)	Nonresponders (n = 20)	*P* value
Age (years)	57.5 ± 10.8	56.0 ± 12.7	0.63
BCVA (logMAR)	0.63 ± 0.36	0.64 ± 0.36	0.85
CMT (μm)	411.3 ± 110.6	459.8 ± 113.5	0.33
HbA1c (%)	7.88 ± 1.31	7.39 ± 1.57	0.29
Duration (years)	12.8 ± 8.2	14.9 ± 9.3	0.49

*BCVA* = best-corrected visual acuity; *CMT* = central macular thickness; *HbA1c* = glycated hemoglobin A1c

The association was estimated using Spearman correlation analysis to determine whether the baseline levels of these cytokines were associated with the response of anti-VEGF therapy. There were significantly positive correlations between baseline VEGF (r_s_ = 0.33, *P* = 0.0072), Ang-2 (r_s_ = 0.39, *P* = 0.001) and response to anti-VEGF therapy (Fig. 3b).

### Prediction model based on baseline cytokine level was constructed to identify the response to anti-VEGF therapy

**Fig. 4:**
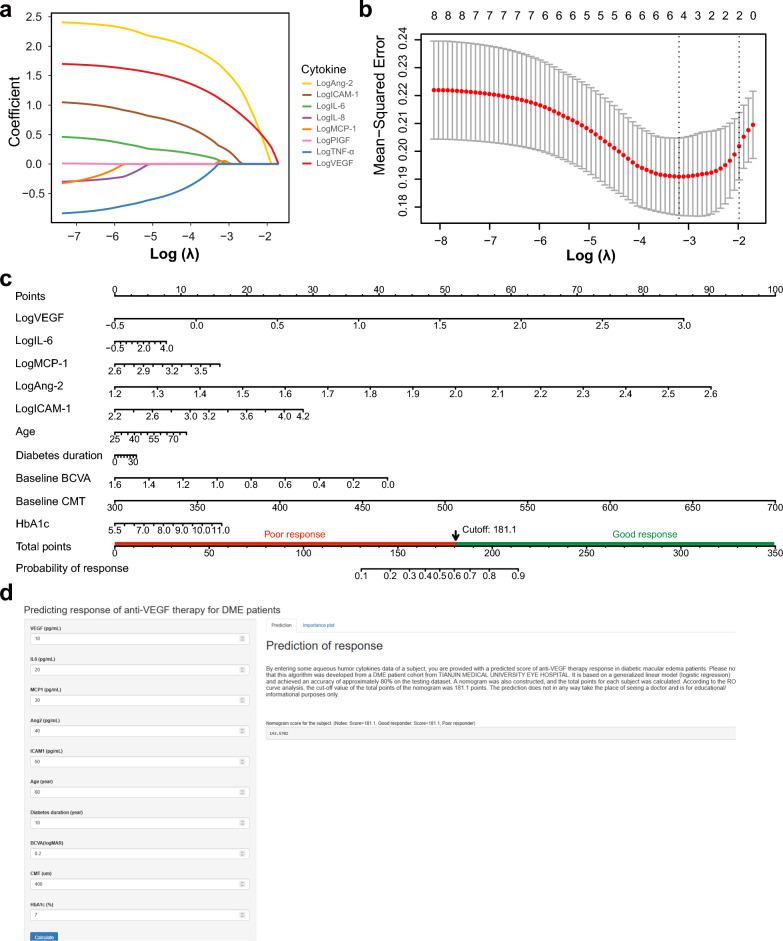
Prediction nomogram model based on baseline cytokine level was constructed to identify the response to anti-VEGF therapy. **a** Features selection using the LASSO regression model. LASSO coefficient profiles of the eight candidate cytokines baseline level. Each curve represents a coefficient, and the x-axis represents the regularization penalty parameter. As λ changes, a coefficient that becomes non-zero enters the LASSO regression model. **b** Selection of the tuning parameter (λ). The left dashed line indicates λ_min, the penalty parameter value that yields the minimum mean cross-validated error. The right dashed line indicates λ_1se, the largest λ value whose error is within one standard error of the minimum. The LASSO logistic regression model was fitted with the penalty parameter (λ) tuned via threefold cross-validation, selecting the value that minimized the cross-validated error (λmin). **c** Nomogram predicting the probability of anti-VEGF therapy in the discovery set. Each variable was assigned a specific score on a rating scale. The scores of each variable were summed, and a vertical line was drawn downward at the location of the total score to obtain the predicted probability of anti-VEGF therapy. Higher total scores indicated a higher probability of response to anti-VEGF therapy. **d** A screenshot for the online nomogram scores calculation. Enter the cytokine concentrations and click the “Calculate” button, the main panel displays the predicted probability of anti-VEGF therapy. The resulting nomogram score is intended to inform individualized patient assessment and clinical decision-making. Ang-2, angiopoietin-2; BCVA, best-corrected visual acuity; CMT, central macular thickness; HbA1c, glycated hemoglobin A1c; ICAM-1, intercellular adhesion molecule-1; IL-6, interleukin-6; IL-8, interleukin-8; MCP-1, monocyte chemoattractant protein-1; TNF-α, tumor necrosis factor-alpha; VEGF, vascular endothelial growth factor

To construct a model for prediction regarding anti-VEGF therapy response, baseline levels of the eight candidate cytokines were used for the least absolute shrinkage and selection operator (LASSO) algorithm analysis. Five factors, including logVEGF, logAng-2, IogIL-6, logMCP-1, and logICAM-1, were selected at the optimal lambda value of 0.041 from the LASSO regression model (Fig. 4a, b). Logistic regression analysis was performed using the selected five factors and clinical covariates including age, duration of diabetes, BCVA, CMT, and HbA1c.

A nomogram with variables logVEGF, logAng-2, IogIL-6, logMCP-1, logICAM-1, and clinical covariates based on the results of the logistic regression analysis were constructed to elaborate the prediction model (Fig. 4c). The total score for each patient was calculated, and an online simple-to-use nomogram (https://cytokine.shinyapps.io/Cytokineapp/) was constructed (Fig. 4d), which can be used as a practical approach for personalized early screening probability of response to anti-VEGF therapy and assist physicians in making a personalized treatment for patients.

**Fig. 5:**
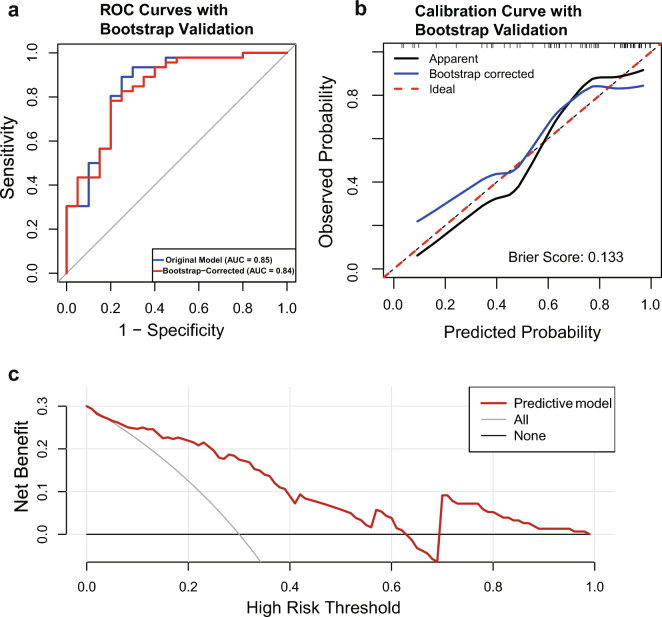
Receiver operating characteristic (ROC) curve and calibration curve for the nomogram. **a** ROC curves for the original model and bootstrap internal validation. **b** Calibration curve of the bootstrap internal validation. The x-axis represents the predicted response probability, and the y-axis represents the actual probability. Ideal: the diagonal dotted line represents a perfect prediction by an ideal model; bootstrap-corrected: the blue solid line represents the performance of the nomogram. The closer the fit to the diagonal dotted line represents a better prediction. **c** Decision curves of the prediction model. All/None: net benefit of the ‘treat all’ or ‘treat none’ strategies. Predictive model: the net clinical benefit if decisions are based on the model. AUC, area under the ROC curve; CI, confidence interval

Using ROC analysis, the prediction model showed good discriminatory ability in the differentiation of response and non-response, with an AUC of 0.85 (95% CI: 0.74–0.96) (Fig. 5a), a specificity of 75% and a sensitivity of 89% was achieved. Using the bootstrap method, the original data was repeatedly sampled 500 times to establish an internal validation set. The risk prediction model achieved an AUC of 0.84 (Fig. 5a) in internal validation, indicating that the model has good discriminative ability. To assess the nomogram’s performance, the calibration curve of the model was generated via the bootstrap internal validation approach (Fig. 5b). The model demonstrated acceptable calibration accuracy, with a Brier score of 0.133, although both calibration curves lay below the ideal diagonal, suggesting a tendency to overestimate risk. A Hosmer–Lemeshow goodness of fit test was performed to assess calibration, yielding a non-significant *P* value of 0.295, which suggests adequate agreement between the model’s predictions and the observed outcomes. Decision curve analysis demonstrated that the predictive model provided a higher net benefit than default clinical strategies (treat-all or treat-none) for threshold probabilities between 0 and approximately 0.22 (Fig. 5c).

Beyond predictive modeling of response using baseline data, we employed survival analysis to evaluate treatment efficacy in the time-to-event dimension, offering insight into the dynamics of view of treatment efficacy. The exploratory Kaplan–Meier analysis revealed a median time to first functional response of approximately 1 month. The cumulative incidence of first response was 53.0% (95% CI: 39.3%–63.7%) at 1 month, 66.7% (95% CI: 53.1%–76.3%) at 3 months, and 69.7% (95% CI: 56.3%–79.0%) at 6 months (Supplementary Fig. S1). This indicates that the majority of functional responses occur within the first 3 months of treatment.

### Different longitudinal cytokine changes were detected in the response- and non-response groups at 1 month versus baseline

**Fig. 6:**
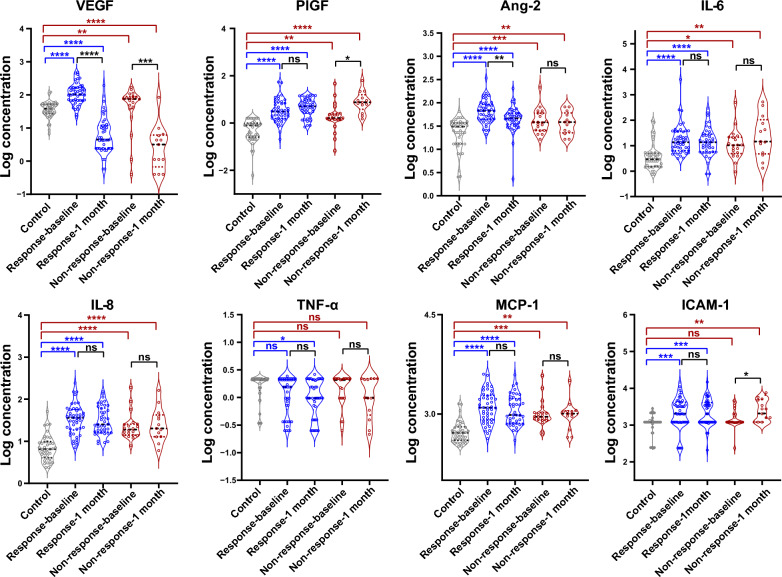
The changes in candidate cytokines at 1 month vs. baseline was compared for the response and non-response groups. For the response group, aqueous at baseline (n = 46) and 1 month (n = 42) were measured. For the non-response group, aqueous at baseline (n = 20) and 1 month (n = 13) were measured. Cytokine data were log-transformed. *P < 0.05, **P < 0.01, ***P < 0.001, ****P < 0.0001, ns, non-significant; Ang-2, angiopoietin-2; ICAM-1, intercellular adhesion molecule-1; IL-6, interleukin-6; IL-8, interleukin-8; MCP-1, monocyte chemoattractant protein-1; PIGF, placenta growth factor; TNF-α, tumor necrosis factor-alpha; VEGF, vascular endothelial growth factor

The expression profiles of candidate cytokines were analyzed to investigate whether the levels of candidate cytokines were changing after the administration of anti-VEGF drugs. In both groups, the level of VEGF was significantly decreased at 1 month vs. baseline (Fig. 6). For the response group, except for VEGF, Ang-2 was also significantly reduced (logAng-2, 1 month vs. baseline, 1.64 ± 0.29 vs. 1.81 ± 0.25, *P* = 0.0064); IL-6, IL-8, and MCP-1 showed a trend in reduction, although they did not reach the conventional statistical significance level (Fig. 6). For the non-response group, PIGF (logPIGF, 1 month vs. baseline, 0.89 ± 0.45 vs. 0.25 ± 0.61, *P* = 0.0012) and ICAM-1 (logICAM-1, 1 month vs. baseline, 3.38 ± 0.28 vs. 3.17 ± 0.27, *P* = 0.0266) was increased on the contrary (Fig. 6). Upon analyzing these findings, we deduced that, for responders, higher levels of VEGF might play a prominent role in the disease, and treatment targeting VEGF can provide relief. However, for non-responders, other factors may contribute to treatment resistance, meaning that reducing VEGF levels alone may not be sufficient to alleviate the disease.

### Level of several cytokines after multiple injections of anti-VEGF drugs

**Fig. 7:**
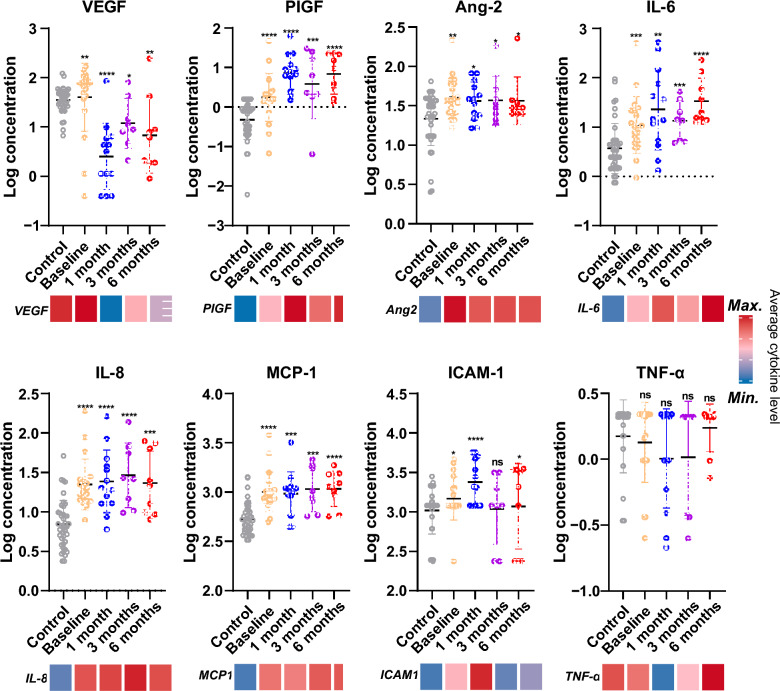
Cytokines were measured in the non-response group at 1, 3, and 6 months post-injection of anti-VEGF drugs. For non-response group, aqueous was collected at baseline (n = 20), and 1 (n = 13), 3 (n = 8), 6 months (n = 9) after injection of anti-VEGF drugs, and the selected cytokines were measured. Cytokine data were log-transformed, and data were presented as mean ± SD. Top, scatter diagram. Bottom, heat map showing the average cytokine level among control and per point. *P < 0.05, **P < 0.01, ***P < 0.001, ****P < 0.0001, ns, non-significant; VEGF, vascular endothelial growth factor; PIGF, placenta growth factor; Ang-2, angiopoietin-2; IL-6, interleukin-6; IL-8, interleukin-8; MCP-1, monocyte chemoattractant protein-1; ICAM-1, intercellular adhesion molecule-1; TNF-α, tumor necrosis factor-alpha

To further investigate the change in expression levels of candidate cytokines in the non-response group, we compared the cytokines level at baseline, 1-month, 3-month, and 6-month visits with the controls. The concentration of VEGF was lower than that of controls post-injection of anti-VEGF drugs (Fig. 7), which may be due to the patients receiving consistent injections of anti-VEGF drugs. However, the cytokines including PIGF, Ang-2, IL-6, IL-8, and MCP-1, were still higher than controls (Fig. 7). Elevated levels were observed in the small subset of patients who completed the 6-month follow-up, which may reflect a subgroup with more sustained disease activity. Due to patients’ refusal to repeat the invasive aqueous humor sampling (MNAR missingness), the long-term trajectory for the entire cohort remains uncertain.

## Discussion

Our objective was to examine the relationship between cytokine levels and the varied response to anti-VEGF treatment. Our research revealed that there are cytokines, other than VEGF, that play a role in the development of DME. We developed a baseline cytokine-based nomogram model that may be used to determine the likelihood of an individual’s response to anti-VEGF medication. This model can assist clinicians in making more informed treatment decisions. Treatment plans that focus on specific cytokines, such as Ang-2, IL-6, and IL-8, might enhance visual clarity and decrease swelling in the macula of patients with DME.

Recently, intravitreal anti-VEGF drugs have been the first-line therapy for DME patients. Although evidence has demonstrated that anti-VEGF therapy can reduce macular edema and improve visual acuity, many DME patients still do not completely respond to the treatment [7]. For the incomplete response, studies have shown that approaches such as increasing the frequency of injections or switching to different anti-VEGF agents, or combining anti-VEGF therapy with other therapeutic regimens, such as corticosteroids can be beneficial [8–11]. While these options exist for non-responders, finding these non-responders early is critical as prolonged treatment can lead to increased risk of side effects and the risk of disease progression, potentially missing optimal treatment opportunities [12–14]. Consequently, the implementation of personalized treatment, early screening strategies, and ongoing research are all essential for the improvement of outcomes for this patient subgroup.

Studies have reported that aqueous cytokines were correlated with the response to anti-VEGF therapy, such as elevated baseline aqueous ICAM-1 and reduced VEGF levels being associated with a favorable anatomic response to ranibizumab in DME [15]; normal VEGF but increased inflammatory cytokines may be insensitive to anti-VEGF treatment [16]; baseline aqueous humor levels of PlGF, MCP-1, and IL-6 were significantly correlated with the improvement in best corrected visual acuity [17]. Compared with single-factor analysis, multi-factor analysis can comprehensively consider the factors influencing the predictor variables and enhance prediction accuracy. Our study aimed to construct a model based on multiple baseline aqueous cytokines to predict the response to anti-VEGF drugs and identify the cytokines beyond VEGF. The findings can assist clinicians in assessing the efficacy of anti-VEGF therapy and formulating more evidence-based treatment strategies. Initially, we selected eight cytokines, including angiogenesis-related cytokines VEGF, PIGF, and Ang-2; inflammatory factor IL-6, IL-8, and TNF-α; chemokine MCP-1, and adhesion factor ICAM-1, that play potential roles in the pathogenesis of DME based on the existing literature [18–20]. Compared to the controls, the selected cytokines, except for TNF-α, increased in the baseline of DME patients. Our results further indicate vascular growth and inflammation-related factors play a role in the initial pathogenesis of DME. Analysis of aqueous humor immune mediators using machine learning has been shown to enable the classification of intraocular diseases and to pinpoint specific biomarkers associated with them [21]. Therefore, predictive models from aqueous cytokine panels may hold the potential for distinguishing between different drug responses. To distinguish between responders and non-responders, a predictive nomogram model was developed using baseline cytokines and clinical covariates, and a simple-to-use online nomogram was also constructed to provide convenience for clinicians. The nomogram showed good discriminative efficiency with AUC values of 0.85 and 0.84 for the discovery and bootstrap inter-validation, respectively. Therefore, a baseline cytokine-based prediction model might differentiate responders from non-responders.

Furthermore, we aimed to explore which factors were affected in non-responders. The study noted no significant difference in baseline aqueous humor VEGF concentration. At the same time, at the 2-month follow-up, the non-responder group had a significantly higher VEGF concentration compared with the responder group, and the responder group also demonstrated a significant decline from baseline to 2-month follow-up in concentration of ICAM-1, IL-10, MCP-1, PIGF, and TGF-β2 [22]; another analysis of the cytokines in the aqueous humor of DME patients showed that super responders had increased baseline VEGF and decreased MCP-1 concentrations compared with non-responders, while IL-6 concentrations increased among non-responders during therapy [23]. Our study found that baseline levels of VEGF, Ang-2 and PIGF were higher in the response group than in the non-response group. Distinct longitudinal cytokine dynamics were observed between responders and non-responders: for the response group, except for decreasing VEGF, Ang-2 was also significantly reduced, and IL-6, IL-8, and MCP-1 tended to decline; for the non-response group, VEGF decreased while PIGF and ICAM-1 increased, and other cytokines were found to have no changes. The results suggest distinct differences in aqueous cytokine change between the response and non-response groups after being given anti-VEGF therapy. Multiple studies demonstrated that VEGF, the critical mediator in DME pathogenesis, was higher in responders than non-responders [23, 24]. Anti-VEGF agents, including ranibizumab, bevacizumab, and aflibercept, directly addressing VEGF overexpression, could reduce retinal vascular permeability and consequently decrease macular edema. For responders with higher VEGF, VEGF plays a dominant role; targeting VEGF agents could decrease macular edema and improve patient visual acuity. For non-responders, other cytokines, such as Ang-2 and/or inflammatory cytokines, might be more dominant than VEGF. Consequently, inhibiting VEGF alone could not achieve a good outcome. We also collected the aqueous from refractory DME patients at 6 months after multiple injections of anti-VEGF drugs; we found that the cytokine VEGF was kept at a lower level, which may be due to the injection of anti-VEGF drugs; the cytokines Ang-2, IL-6, IL-8, MCP-1, and PIGF were higher in a small subset of patients who completed the 6-month follow-up. However, due to patients’ refusal to repeat the invasive aqueous humor sampling, the long-term trajectory for the entire cohort remains uncertain.

Ang-2 has been reported to be markedly increased in the vitreous of DME patients [25]. Increased Ang-2 can stimulate the phosphorylation and degradation of VE-cadherin, an endothelial-specific adhesion molecule, which increases of vascular permeability, and thus promotes macular edema [26]. In addition to the influence of vascular integrity, Ang-2 also has effects on pro-inflammatory factors, including macrophage polarization and neutrophil infiltration [27, 28]. Furthermore, preclinical data demonstrated that the inhibition of Ang-2 could reduce inflammation and increase vascular stability [29, 30]. Faricimab, a bispecific antibody, can bind to and deplete VEGFA and Ang-2, which maintains vascular stability and integrity. The intravitreal injection of faricimab has shown promising results in clinical trials among patients with neovascular age-related macular degeneration (nAMD) and DME, and faricimab has since received its first approvals for nAMD and DME in the USA. In our non-response group, the level of Ang-2 was still upregulated at 1 and 6 months after receiving anti-VEGF, indicating that therapy targeting Ang-2 may improve the treatment effect for refractory DME patients. Given this observational correlation, it is necessary to analyze aqueous humor from patients who switch to faricimab, thereby linking biomarker changes to clinical response and providing mechanistic validation.

IL-6 belongs to the pro-inflammatory cytokine family. As a master player in the cytokine network, IL-6 effects several immune and physiological processes [31]. IL-6 is a critical mediator in inflammation, autoimmunity, and cancer, with its primary effects orchestrated through the IL-6-signal transducer and activator of signal transducer and activator of transcription 3 (STAT3) pathway [32, 33]. Aqueous IL-6 was identified to be associated with macular thickness in patients with AMD and DME [34, 35]. IL-6 can disrupt the integrity of the blood-retinal barrier, leading to macula edema. In an animal model, intravitreal injection of IL-6 antagonism can reduce choroidal neovascularization. IL-8, a member of the CXC family of chemokines, is a proinflammatory chemokine. Under inflammatory conditions, the produced IL-8 can attract neutrophils to the inflammation sites [36]. The activity of CXCL8 is intrinsically dependent on its binding to the human CXC chemokine receptors CXCR1 and CXCR2, the atypical chemokine receptor ACKR1, and glycosaminoglycans [37]. In a murine model, a CXCR1/CXCR2 antagonist prevented and reversed type 1 diabetes [38]. Compared to DME patients without subretinal fluid, the vitreous IL-8 level was measured to be significantly upregulated in DME patients with subretinal fluid, which indicates that IL-8 plays an important role in the formation of subretinal fluid participating in the progression of DME [39]. MCP-1, a member of CC chemokines, plays an essential role in the inflammatory process and can attract or promote the expression of other inflammatory factors. MCP-1 was identified to be strikingly higher in diabetic patients with PDR and to be associated with PDR activity [40]. MCP-1 is involved in the progression of various disorders by activating its receptor CCR2 and signaling pathways (e.g., Akt signaling pathway, ERK pathway) [41–43]. The level of MCP-1 was significantly higher in patients with DME than both in nondiabetic patients and diabetic patients without retinopathy [44, 45]. In our study, IL-6, IL-8, and MCP-1 were still significantly higher at 6 months after multiple injections of anti-VEGF drugs. These results are consistent with previous studies indicating that inflammatory factors play a role in DME. Their study reported that in DME patients who received intravitreal injections of triamcinolone, IL-6 and MCP-1 were significantly decreased, and there was more of a reduction in foveal thickness as compared to the anti-VEGF drug treatment group [46, 47]. Another study reported that a combination of intravitreal anti-VEGF drug and triamcinolone injection for DME patients can reduce the level of MCP-1 but increase the IL-8 level [48]. From the existing literature and our study’s findings, there seem to be other cytokines, beyond VEGF, participating in the pathogenesis and progression of DME. Hence, therapeutic strategies targeting inflammatory pathways may offer benefit in the management of DME. However, further studies, such as profiling the aqueous humor of anti-VEGF non-responders who are transitioned to steroid-containing regimens, are warranted to inform the rational selection of anti-inflammatory agents.

The limitations of our study are as follows. Initially, our investigation was conducted retrospectively, and the width of the confidence intervals for the AUC estimates reflects the precision limitations inherent to the current sample size. Consequently, it would be advisable to conduct a future multicenter clinical trial employing an external validation in a larger, independent multicenter cohort to validate the therapeutic advantages. Secondly, the prediction model relied on a certain set of cytokines that were chosen. However, there are additional cytokines that also impact the development of DME. Therefore, it is possible to continually select relevant cytokines in the future to enhance the accuracy of the prediction model. Thirdly, our records pertaining to hypertension status and glucose-lowering medication regimens are characterized by incomplete coverage. The absence of these critical clinical variables represents a notable weakness of our study model; thus, systematic collection of these data in subsequent studies will be essential to achieve more precise risk stratification and robust mechanistic exploration. Finally, in our study, while the definition of treatment response was based on clinically conventional BCVA thresholds, this approach is inherently arbitrary, and future research should aim to establish more objective and validated criteria, as differing thresholds can alter the proportion of patients classified as responders.

## Conclusions

Our data demonstrated that a baseline cytokine-based prediction model might be used to differentiate responders and non-responders. Other cytokines, e.g., Ang-2, IL-6, IL-8, and MCP-1, were significantly increased in non-responsive DME patients. Therapeutic regimens targeting these cytokines may improve visual acuity and reduce retinal thickness in patients with DME.

## Supplementary Information

Additional file 1.

## Data Availability

The data used to support this study’s findings is available upon reasonable request to the corresponding author.

## Abbreviations

DME: Diabetic macular edema
DR: Diabetic retinopathy
CMT: Central macular thickness
OCT: Optical coherence tomography
BCVA: Best-corrected visual acuity
IOP: Intraocular pressure
ROC: Receiver operating characteristic
AUC: Area under the receiver operating characteristic curve
