# Supplementary material for: Development of a predictive model based on aqueous cytokines data for response to anti-VEGF therapy in diabetic macular edema
Source: Eye Vis (Lond). 2026 Mar 13;13:12. doi: 10.1186/s40662-026-00479-z (PMC12983647; doi:10.1186/s40662-026-00479-z)
Supplement: Supplementary file 1 — Additional file 1. [file 40662_2026_479_MOESM1_ESM.docx]

**Supplementary Materials**

**Supplementary Figure 1.** Kaplan‑Meier survival curves show the cumulative probability of patients remaining non‑responders over time.


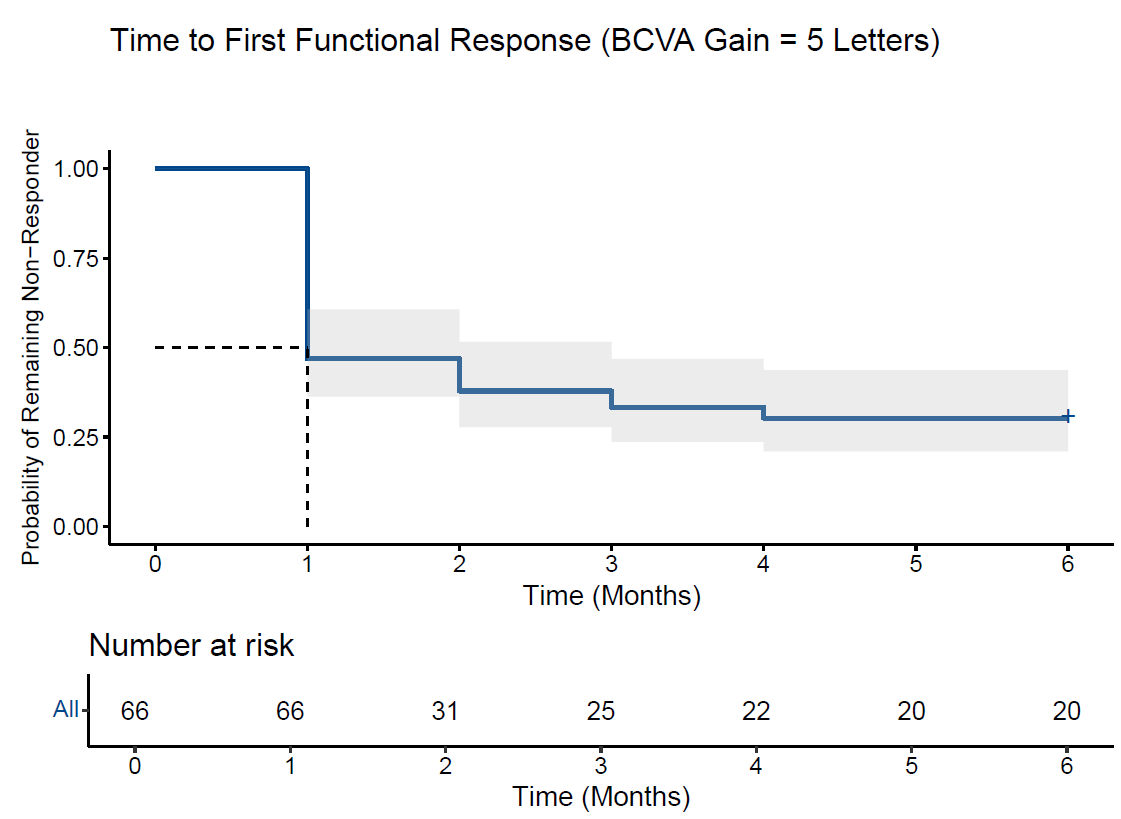


**Supplementary Table S1**. List of cytokines analyzed by the Luminex panel.

| **Cytokine** | **Standard curve range (pg/mL)** | **Sensitivity (pg/mL)** | **Inter-assay CVs** |
| --- | --- | --- | --- |
| **VEGF** | 11.5–2800 | 0.99 | 6.5% |
| **TNF-α** | 8.23–2000 | 1.2 | 15.9% |
| **IL-6** | 3.25–790 | 1.7 | 3.3% |
| **IL-8** | 4.12–1000 | 1.8 | 7.8% |
| **MCP-1** | 30.9–7500 | 9.9 | 7.0% |
| **Ang-2** | 94.7–23000 | 17.1 | 8.2% |
| **ICAM-1** | 3200–775,000 | 87.9 | 4.7% |
| **PIGF** | 2.88–700 | 1.9 | 6.7% |

Ang-2 = angiopoietin-2; CV = coefficient of variation; ICAM-1 = intercellular adhesion molecule-1; IL-6 = interleukin-6; IL-8 = interleukin-8; MCP-1 = monocyte chemoattractant protein-1; PIGF = placenta growth factor; TNF-α = tumor necrosis factor-alpha; VEGF = vascular endothelial growth factor

**Supplementary Table S2.** Comparison of baseline cytokine levels in patients with diabetic macular edema (DME) versus control patients with cataract.

| **Cytokine** | **DME**  **(n = 85)**  **Median (pg/mL)** | **Control**  **(n = 41)**  **Median (pg/mL)** | **Median Difference** | **Log(DME)**  **Median (mean ± SD)** | **Log(Control)**  **Median (mean ± SD)** | ***P* value** | **Adjusted**  ***P* value** |
| --- | --- | --- | --- | --- | --- | --- | --- |
| VEGF | 89.9 | 38.5 | 51.4 | 1.9 (1.9 ± 0.5) | 1.5 (1.5 ± 0.3) | 2.65E−09 | 4.25E−09 |
| PIGF | 2.2 | 0.8 | 1.4 | 0.3 (0.4 ± 0.5) | −0.1 (−0.3 ± 0.5) | 1.55E−11 | 3.10E−11 |
| Ang2 | 66.2 | 30.8 | 35.4 | 1.8 (1.8 ± 0.3) | 1.5 (1.3 ± 0.3) | 1.36E−11 | 3.10E−11 |
| IL-6 | 11.3 | 2.9 | 8.4 | 1.1 (1.2 ± 0.6) | 0.5 (0.6 ± 0.5) | 1.53E−08 | 2.04E−08 |
| IL-8 | 27.9 | 6.5 | 21.4 | 1.2 (1.5 ± 0.4) | 0.8 (0.8 ± 0.3) | 1.07E−16 | 1.71E−15 |
| MCP1 | 1003.1 | 518.7 | 514.4 | 3.0 (3.1 ± 0.2) | 2.7 (2.7 ± 0.2) | 1.85E−12 | 7.41E−12 |
| ICAM1 | 1973.9 | 1203.9 | 770.0 | 3.3 (3.3 ± 0.4) | 3.1 (3.0 ± 0.3) | 5.40E−04 | 6.18E−04 |
| TNF-α | 2.1 | 2.1 | 0 | 0.3 (0.1 ± 0.3) | 0.3 (0.2 ± 0.3) | 0.769 | 0.769 |

Ang-2 = angiopoietin-2; ICAM-1 = intercellular adhesion molecule-1; IL-6 = interleukin-6; IL-8 = interleukin-8; MCP-1 = monocyte chemoattractant protein-1; PIGF = placenta growth factor; SD = standard deviation; TNF-α = tumor necrosis factor-alpha; VEGF = vascular endothelial growth factor

*P* values were adjusted for multiple comparisons across all cytokines tested using the Benjamini-Hochberg false discovery rate (FDR) method.

**Supplementary Table S3.** Comparison of baseline cytokine levels in response versus non-response group.

| **Cytokine** | **Responders**  **(n = 46)**  **Median (pg/mL)** | **Non-responders**  **(n = 20)**  **Median (pg/mL)** | **Median Difference** | **Log(DME)**  **Median (mean ± SD)** | **Log(Control)**  **Median (mean ± SD)** | ***P* value** | **Adjusted**  ***P* value** |
| --- | --- | --- | --- | --- | --- | --- | --- |
| VEGF | 103.9 | 75.4 | 28.5 | 2.0 (2.0 ± 0.3) | 1.8 (1.6 ± 0.7) | 0.0075 | 0.0300 |
| PIGF | 3.0 | 1.6 | 1.4 | 0.5 (0.5 ± 0.5) | 0.2 (0.2 ± 0.6) | 0.0498 | 0.0797 |
| Ang2 | 67.1 | 37.9 | 29.2 | 1.8 (1.8 ± 0.2) | 1.6 (1.6 ± 0.3) | 0.0012 | 0.0096 |
| IL-6 | 13.6 | 10.4 | 3.2 | 1.1 (1.2 ± 0.6) | 1.0 (1.0 ± 0.6) | 0.2047 | 0.2319 |
| IL-8 | 36.2 | 19.0 | 17.2 | 1.6 (1.5 ± 0.4) | 1.3 (1.3 ± 0.3) | 0.0446 | 0.0797 |
| MCP1 | 1250.0 | 909.2 | 340.8 | 3.1 (3.1 ± 0.2) | 3.0 (3.0 ± 0.2) | 0.0734 | 0.0979 |
| ICAM1 | 2050.1 | 1203.9 | 846.2 | 3.3 (3.3 ± 0.4) | 3.1 (3.2 ± 0.3) | 0.0286 | 0.0762 |
| TNF-α | 1.5 | 2.1 | -0.6 | 0.2 (0.05 ± 0.3) | 0.3 (0.1 ± 0.3) | 0.2319 | 0.2320 |

Ang-2 = angiopoietin-2; DME = diabetic macular edema; ICAM-1 = intercellular adhesion molecule-1; IL-6 = interleukin-6; IL-8 = interleukin-8; MCP-1 = monocyte chemoattractant protein-1; PIGF = placenta growth factor; SD = standard deviation; TNF-α = tumor necrosis factor-alpha; VEGF = vascular endothelial growth factor

*P* values were adjusted for multiple comparisons across all cytokines tested using the Benjamini-Hochberg false discovery rate (FDR) method.
